# Diagnosis and treatment of vulnerable migrants: a retrospective study at a Doctors of the World clinic in Stockholm

**DOI:** 10.1186/s12913-021-07410-3

**Published:** 2022-02-17

**Authors:** Klas Ytterbrink Nordenskiöld, Jan-Eric Olsson, Bo C Bertilson

**Affiliations:** 1 Academic Primary Care Center, Stockholm Health Care Services (SLSO), Solnavägen 1 E, 11365 Stockholm, Sweden; 2 Doctors of the World Sweden, Hantverkargatan 2c, 11221 Stockholm, Sweden; 3 Academic Primary Care Center, Stockholm Health Care Services (SLSO), Solnavägen 1 E, 11365 Stockholm, Sweden; 4 Department of Neurobiology, Care Sciences and Society, Karolinska Institutet, Alfred Nobels allé 23, 141 83 Huddinge, Sweden

**Keywords:** Vulnerable populations, Transients and migrants, Diagnoses, Therapeutics, Retrospective studies

## Abstract

**Background:**

At Doctors of the World Medical Clinic in Stockholm (DWMCS), medical care is offered to migrants who live under particularly vulnerable conditions and who lack access to subsidized care. The demographic, diagnostic and therapeutic panorama of vulnerable migrants is unknown.

**Methods:**

A quantitative, retrospective study mapping gender, age, diagnostic group, primary diagnosis, therapeutics, referrals, and session timing (whether the care session took place in summer -April to September, or winter - October to March) by reading all patients’ electronic journals at DWMCS between 2014-04-01 and 2017-12-31. Diagnostic groups were classified according to the classification system ICPC-2 which contains six diagnostic groups: symptoms/complaints, infections, neoplasms, injuries, congenital anomalies and other diagnoses. Primary diagnosis was defined as the diagnosis that was first in the diagnosis list for the visit. Difference in median age was calculated with the Mann-Whitney test (MW), and two-group analysis of nominal data was performed with Monte Carlo simulations (MC) and chi square test´s (X^2^).

**Results:**

The study included 1323 patients: 838 women and 485 men. The median age for women 37 years (29-47) was slightly lower than for men, 40 years (31-47) MW (p = 0.002). The largest diagnostic group was symptoms / complaints. The five most common primary diagnoses were cough (4%), back symptom / complaint (4%), cystitis (3%), upper respiratory infection acute (3%) and abdominal pain epigastric (2%). The most common therapeutic (55%) was pharmaceutical. Referrals accounted for 12% of the therapeutics and 25% of the referrals were to an emergency room. Tests of significance indicated an uneven distribution of diagnostic groups MC (p = 0.003), infectious primary diagnoses MC (p = 0.0001) and referrals MC (p = 0.006) between men and women and an uneven seasonal distribution among the Other diagnoses MC (0.04) and ten most common drug treatments MC (p=0.002).

**Conclusions:**

The demographic, diagnostic and therapeutic panorama of vulnerable migrants at DWMCS was elucidated. Vulnerable migrants have differences in morbidity depending on gender and season, differences in therapeutics depending on gender and differences among their most common drug treatments depending on season. This knowledge is important when addressing the health problems of vulnerable migrants.

## Background

Vulnerable populations are defined as groups of persons whose range of options is severely limited, who are frequently subjected to coercion in their decision making, or who may be compromised in their ability to give informed consent [1]. Vulnerable populations have increased risk of disease [2], increased risk of not having their care needs met [3] and increased risk of premature death [4].

Migrants are by definition people who change their country of usual residence [5]. The term includes people who move between as well as within nations and is independent of whether they move permanently or temporarily [6]. In 2014, more than 200 million migrants moved between countries - more than 3% of the world’s population [7] and in Sweden there were more than 1.6 million migrants born abroad [8]. Migrants often have reduced access to health care due to legal restrictions on access to care, lack of health insurance, insufficient funds to pay fees, cultural misunderstandings, and linguistic problems [9].

Diagnoses may be differently distributed between migrants compared to domestic population. Migrants may, for example, have a higher incidence of tuberculosis [10] and have increased mortality related to infectious diseases [11]. Therapeutics of infectious diseases may be delayed for migrants [12] and the mental health problems of undocumented migrants may be undertreated in primary care [13]. Migrants have an increased risk associated with pregnancy [14] and lower use of contraceptives in connection with abortion [15]. The most common diagnoses in regular primary care in Stockholm are acute upper respiratory tract infection, essential hypertension, cough and back pain [16].

Since 1995, Doctors of the World has run a medical outpatient clinic in Stockholm. Patients who are offered care at Doctors of the World’s Medical Clinic in Stockholm (DWMCS) are migrants, live in Sweden under particularly vulnerable conditions, often have a markedly limited room for maneuver, may lack literacy, and often have a history of exposure to violence. It is unknown how the patient group is composed in terms of gender and age, what diagnoses they have, what therapeutics they receive, where they are referred to, and whether there are differences in morbidity and therapeutics depending on gender and season (summer and winter). The aim of this study was to explore these knowledge gaps about vulnerable migrants and to provide new knowledge about the health problems of this vulnerable population which, by not being able to seek regular subsidized care, go under the radar of Swedish health authorities and are excluded from the Swedish health data register.

## Methods

This is a quantitative, retrospective study of medical records within the framework of existing resources at DWMCS. We mapped gender, age, seasonal distribution for the medical visits, diagnostic groups, thirty most common primary diagnoses, therapeutics, ten most common drug treatments and referrals by studying all patients’ electronic records on DWMCS between 2014-04-01 and 2017-12-31. The time interval was determined based on the date when DWMCS first began using a computerized medical record system until the start of the study. Ethnicity and country of origin were not included because they were judged to be sensitive information andunreliable, and they may increase the possibility of identifying individual study participants. The medical record system at DWMCS was ProRenata version 2.142.0.

The research subjects consisted of all patients at the clinic who had an electronic medical record. Inclusion criteria were that there was a medical record note with age, date, gender and diagnosis according to the diagnosis manual ICPC-2. Exclusion criteria were whether there was a double record for the same patient or if the birth data for a record was filled in incorrectly. There were 796 patients (38%) excluded: 547 (69%) women, 240 (30%) men and 9 (1%) without a stated gender. The reasons for exclusion were lack of diagnosis (85.3%), no reception note (4.1%), dentist note without diagnosis (2.6%), double medical record (2.6%), incorrect birth data (2.1%), deleted or missing journal (1.5%), missing gender information (1.1%) and exercise journal (0.5%).

### Data gathering

Patient data were extracted from the medical records by manual reading, entered into an Excel matrix and processed after anonymization. Anonymization took place using a code key. The code key was created by separating the subjects’ names and birth data from the other data in a separate Excel file after they were assigned a serial number. The code key was saved in a password-protected file on a USB memory stick. The serial numbers were also saved together with the data that was extracted and processed.

If there were several visits, only the first visit that met the inclusion criteria was included. If there were several diagnoses, only the primary diagnosis was included, i.e. the diagnosis that was first in the diagnosis list for the visit. The primary diagnoses were grouped according to ICPC-2 into either Symptoms / Complaints, Other diagnoses, Infections, Injuries, Tumors or Congenital malformations. If there was a defined therapeutic for the primary diagnosis, this was included. If there were several therapeutics indicated for the primary diagnosis, only the first mentioned was included. The therapeutics were grouped into either Pharmaceutical, Referral, Other treatment or No treatment. Pharmaceutical treatments were classified to a therapeutic subgroup according to the second level of the Anatomical Therapeutic Chemical Classification System (ATC). Referrals were categorized depending on the recipient to Health centers, Emergency rooms, Abortion clinics and Other recipients. Other recipients included dentists, maternity care, infection clinics and opticians. Due to regulations in the Swedish Health and Medical Care Act, follow-up information on primary diagnosis from referral clinics could not be collected.

### Statistical methods

Microsoft Excel version 16.16.18 was used for descriptive statistics and structuring of tables. Nominal data such as diagnostic groups, diagnoses, therapeutics, drug treatments and referrals were compiled as pivot tables. The size ratio between the number of patients who were women and the number who were men was described as a gender ratio by dividing the number of women by the number of men. The size ratio between the number of patients who sought help during the summer (April to September) and the winter (October to March) was described as a seasonal ratio by dividing the number of patients during the summer by the number of patients during the winter. Age as interval data was not normally distributed, the difference in median age was chosen as a comparative measure and calculated with the Mann-Whitney test. Two-group analysis of nominal data was performed with Monte Carlo simulations (MC) and chi square test´s (X^2^). MC and X^2^ requires independence of observations why only one outcome for each category of nominal data could be included for every patient. MC was chosen for analyses where there were outcomes with less than five patients and X^2^ was chosen when all outcomes had at least five patients. For two-group analysis of nominal data, outcomes with a very small proportion of total outcomes (<1%) were excluded and outcomes only possible for one gender, for example pregnancy and abortion, excluded that specific outcome. For all statistical calculations, the statistics program Past 3 version 3.24 was used. The significance level was determined to be 5% (p <0.05).

## Results

During the period of 2014-04-01 to 2017-12-31, 2119 patients were registered in DWMCS´s medical record system. Inclusion criteria were met by 1323 (62%) of these, of which 838 were women and 485 were men. The median age of women was 37.3 (28.9-46.6) years and 39.9 (30.6-47.3) years for men; tests of significance indicates that these differed significantly MW (p = 0.02). Gender, seasonal and age group distribution for all patients are shown in Table 1.

**Table 1:**
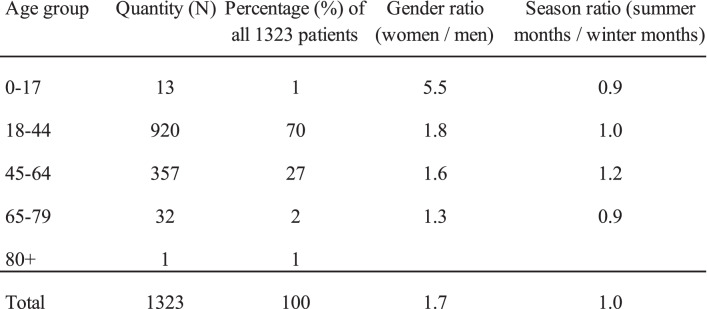
Gender, seasonal and age group distribution for all patients

The largest diagnostic group were symptoms / complaints with more than half of all diagnoses. Tests of significance indicate that the diagnostic groups are not evenly distributed between men and women, MC (p = 0.003). Tests of significance indicated no difference in the seasonal distribution for the diagnostic groups, MC (p = 0.49). Gender and seasonal distribution for the diagnostic groups are shown in Table 2.

**Table 2:**
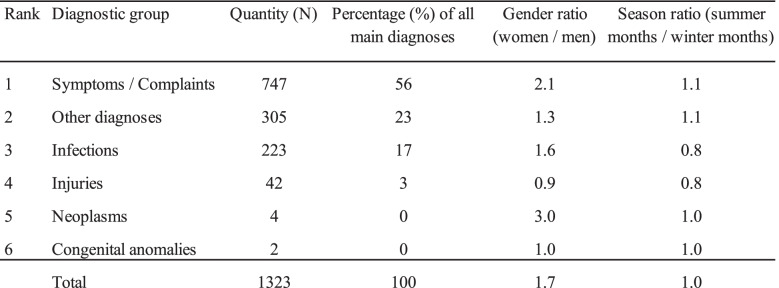
Gender and seasonal distribution for the diagnostic groups

The thirty most common primary diagnoses accounted for 47% of all primary diagnoses. Tests of significance indicate that the distribution of infectious primary diagnoses are not evenly distributed between men and women MC (p = 0,0001) and that the seasonal distribution of the most common Other diagnoses differed significantly MC (p = 0.04), not including pregnancy. Tests of significance indicated no difference in the gender based distribution for the most common Symtoms/Complaints, Other diagnoses or seasonal based distribution of Symtoms/Complaints and Infections, MC (p = 0.5) MC (p = 0.6) MC (p = 0.09) MC (p = 1.0). Gender and seasonal distribution for the thirty most common primary diagnoses, reported for each diagnostic group with p-values calculated with MC are shown in Table 3a-b.

**Table 3:**
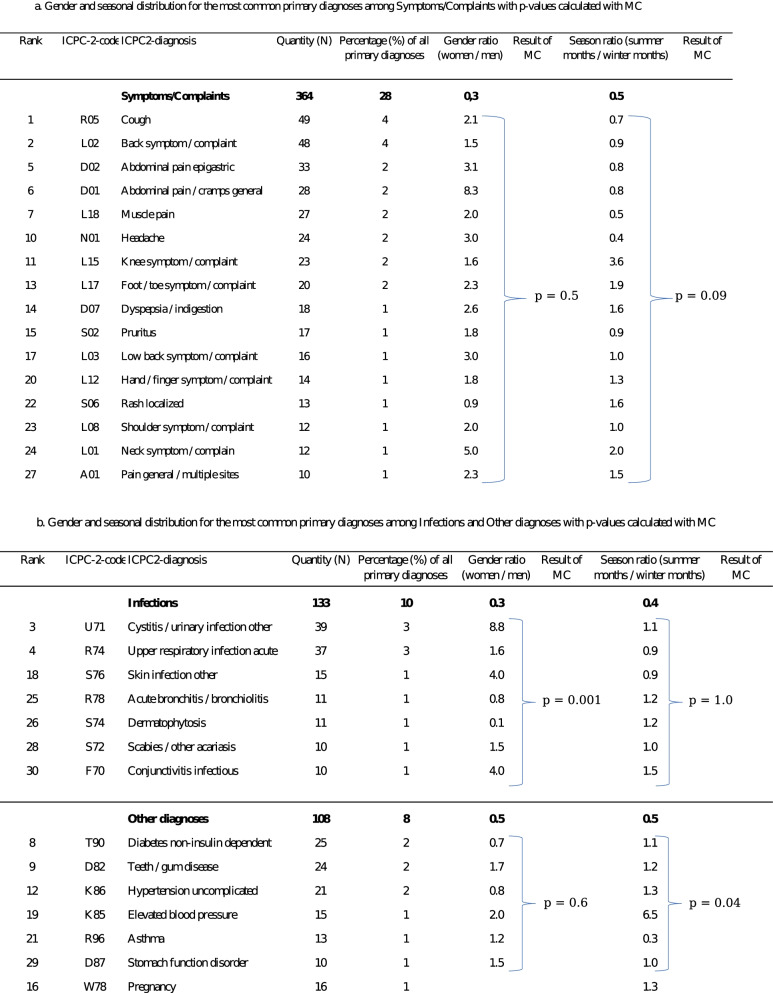
a-b Gender and seasonal distribution for the thirty most common primary diagnoses, reported for each diagnostic group with p-values calculated with MC

Tests of significance indicated no difference in the gender based or seasonal distribution for therapeutics, X^2^ (p = 0.7) and X^2^ (p = 0.8). Gender and seasonal distribution for the therapeutics are shown in Table 4.

**Table 4:**
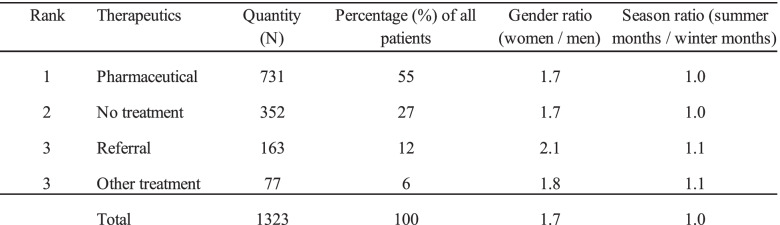
Gender and seasonal distribution for the therapeutics

The ten most common drug treatments corresponded to 75% of all drug treatments. Tests of significance indicated no difference in the gender based distribution for the ten most common drug treatments, X^2^ (p = 0.06). Tests of significance indicates that the seasonal distribution for the ten most common drug treatments differed significantly, MC (p = 0.003). Gender and seasonal distribution for the ten most common drug treatments are shown in Table 5.

**Table 5:**
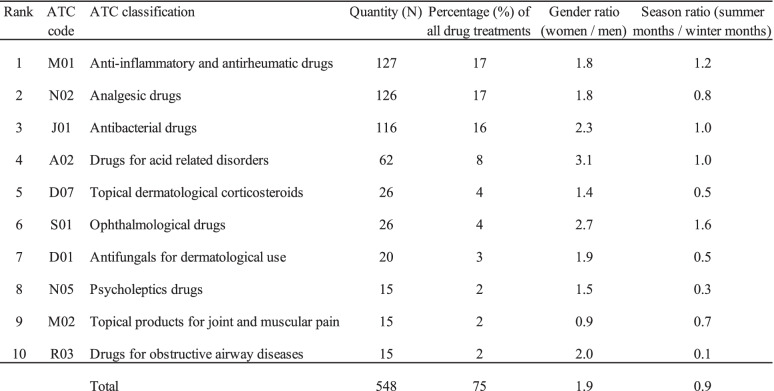
Gender and seasonal distribution for the ten most common drug treatments

Referrals accounted for 12.3% of all treatments. Dentists, maternity care, infection clinics and opticians were summarized as “Other recipients”. Tests of significance indicates that the distribution of referrals differed significantly between men and women, X^2^ (p = 0.02) not including abortion. Tests of significance indicated no difference in the seasonal distribution of referrals, MC (p = 0.3). Gender and seasonal distribution for referrals are shown in Table 6.

**Table 6:**
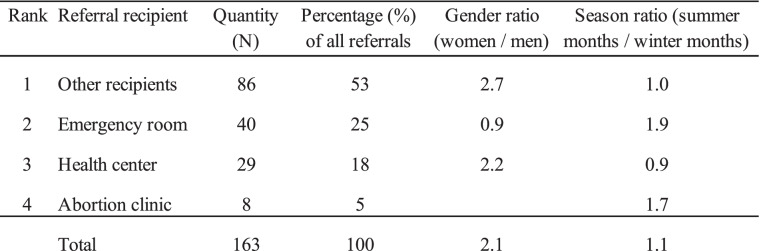
Gender and seasonal distribution for referrals

## Discussion

DWMCS’s patients are dominated by women aged 18-44 years. Cough, pain, uncomplicated infections, adult-onset diabetes and high blood pressure make up a significant proportion of vulnerable migrants’ primary diagnoses. The majority of diagnoses are more common among women.

In this study, the age group 18-44 years accounted for 70% while there were very few children and elderly. This differs considerably from the general primary care population in Stockholm where the age group 80+ years is the smallest with 13% and the age group 18-44 years is the biggest with 26% [16]. It also differs from the age group distribution of Sweden’s total migrant population in 2014 where 80+ years was the smallest with 3% and 18-44 years the biggest with 47% [8]. The fact that children appear at DWMCS is remarkable because children, regardless of legal status, should have the right to subsidized healthcare in Sweden. This is in accordance with what has been shown in previous research: that it is more difficult for migrants than natives to receive medical care [9].

The thirty most common diagnoses for vulnerable migrants described according to ICPC-2 are similar to the thirty most common diagnoses for all of Stockholm’s primary care patients according to ICD-10. Upper respiratory tract infection, cystitis, back and abdominal pain are among the most common. Diabetes and high blood pressure are present in both populations. Depression and anxiety are found as the eighteenth and twenty-sixth most common diagnosis in Stockholm’s primary care [16] but are not among the thirty most common main diagnoses at DWMCS. Previous studies have shown that undocumented migrants’ mental health problems can be undertreated in primary care [13]. The results of this study raise questions about how symptoms of depression and anxiety are expressed by vulnerable migrants, and how this is captured by physicians at the clinic.

Risks associated with pregnancy are increased for migrants [14]. It is less common among migrants than natives that contraceptives are planned to be used after an abortion [15]. In this study, pregnancy was the sixteenth most common primary diagnosis and half of the pregnancies were referred to an abortion clinic. This gives an abortion ratio of 50% which is considerably higher than the overall Swedish abortion ratio in 2014 which was 24% [17, 18].

A limitation of this study is that it only examined the primary diagnosis for the first visit. At an early stage of investigation, symptom diagnoses become a necessity while waiting for investigation results. It is therefore unclear whether a clearer etiological picture of the health problems could have emerged with a method that included all care visits. The clinic’s limited resources when it comes to laboratory analyzes and X-ray examinations may also have shifted the diagnostic panorama towards more symptom diagnoses, as the etiology has been difficult to determine. The degree of exclusion was 37.6%, primarily due to lack of a primary diagnosis in the medical records, this means that the results must be interpreted with caution.

## Conclusions

This study offers an insight into the demographic, diagnostic and therapeutic panorama of vulnerable migrants at DWMCS. Vulnerable migrants have differences in morbidity and therapeutics depending on gender. There is a seasonal difference among the most common drug treatments. This knowledge is important when aiming at using the resources of DWMCS in an effective way when offering free health care to vulnerable migrants. The knowledge is also important for the training of new health workers at Doctors of the World Clinics in Sweden and Europe, and in the communication with Swedish health authority’s when addressing the health problems of this vulnerable population. Migration is an ever-current issue in European societies and new knowledge is constantly needed to follow the development of health problems for migrants.

## Data Availability

Data cannot be shared publicly. This is due to the following reasons. 1.1. The dataset contains too many indirect identifiers: Gender, age, time-interval for the study, diagnosis and therapeutics.2.2. The study population is considered a vulnerable population, so extra care needs to be shown to their integrity.3.3. The study population is relatively small while the risk of being able to identify a participant is increased. This was concluded in dialog with the Swedish national data service https://snd.gu.se/en who states that the data set cannot at all be handled by them. Data are available on request from the authors for researchers who meet the criteria for access to confidential data.

## Abbreviations

DWMCS: Doctors of the World Medical Clinic in Stockholm
ATC: Anatomical Therapeutic Chemical Classification System
MC: Monte Carlo simulations
X^2^: chi square test's
MW: Mann-Whitney test
